# Minor head trauma in infants — how accurate is cranial ultrasound performed by trained radiologists?

**DOI:** 10.1007/s00431-023-04939-9

**Published:** 2023-04-24

**Authors:** Claudia Peter, Enno Stranzinger, Johannes T. Heverhagen, Kristina Keitel, Fabrizio Romano, Jasmin D. Busch, Nedelina Slavova

**Affiliations:** 1 Department of Diagnostic, Interventional and Pediatric Radiology, Inselspital, Bern University Hospital, University of Bern, Bern, Switzerland; 2 Division of Paediatric Emergency Medicine, Department of Paediatrics, Inselspital, Bern University Hospital, University of Bern, Bern, Switzerland; 3 Pediatric Radiology, University Children’s Hospital (UKBB) Basel and University of Basel, Basel, Switzerland; 4 Institute of Diagnostic and Interventional Neuroradiology, Inselspital, Bern University Hospital, University of Bern, Bern, Switzerland

**Keywords:** Cranial ultrasound, Traumatic brain injury, Head trauma, Infants, Intracranial hemorrhage

## Abstract

Correct management of infants after minor head trauma is crucial to minimize the risk to miss clinically important traumatic brain injury (ciTBI). Current practices typically involve CT or in-hospital surveillance. Cranial ultrasound (CUS) provides a radiation-free and fast alternative. This study examines the accuracy of radiologist-performed CUS to detect skull fracture (SF) and/or intracranial hemorrhage (ICH). An inconspicuous CUS followed by an uneventful clinical course would allow exclusion of ciTBI with a great certainty. This monocentric, retrospective, observational study analyzed CUS in infants (< 12 months) after minor head trauma at Bern University Children’s Hospital, between 7/2013 and 8/2020. The primary outcome was the sensitivity and specificity of CUS in detecting SF and/or ICH by comparison to the clinical course and to additional neuroimaging. Out of a total of 325 patients, 73% (*n* = 241) had a normal CUS, 17% (*n* = 54) were found with SF, and ICH was diagnosed in 2.2% patients (*n* = 7). Two patients needed neurosurgery and three patients deteriorated clinically during surveillance. Additional imaging was performed in 35 patients. The sensitivity of CUS was 93% ([0.83, 0.97] 95% CI) and the specificity 98% ([0.95, 0.99] 95% CI). All false-negative cases originated in missed SF without clinical deterioration; no ICH was missed.

*  Conclusion*: This study shows high accuracy of CUS in exclusion of SF and ICH, which can cause ciTBI. Therefore, CUS offers a reliable method of neuroimaging in infants after minor head trauma and gives reassurance to reduce the duration of in-hospital surveillance.

**Table:** 

**What is Known:**
*• Minor head trauma can cause clinically important traumatic brain injury in infants, and the management of these cases is a challenge for the treating physician. *
*• Cranial ultrasound (CUS) is regularly used in neonatology, but its accuracy after head trauma in infants is controversial. *
**What is New:**
*• CUS performed by a trained radiologist can exclude findings related to clinically important traumatic brain injury (ciTBI) with high sensitivity and specificity. It therefore offers reassurance in the management of infants after minor head trauma.*

**Supplementary Information:**

The online version contains supplementary material available at 10.1007/s00431-023-04939-9.

## Introduction

Head trauma is a common reason for emergency department (ED) visits of infants [1, 2]. Most infants show no or only minor symptoms and recover without consequences [3, 4]. However, 3–10% of infants with apparently minor blunt head trauma have traumatic brain injury (TBI) such as intracranial hemorrhage (ICH) or skull fracture (SF) on neuroimaging [3]. One percent of them even have clinically important TBI (ciTBI) requiring prolonged intensive care and/or neurosurgical intervention (as defined in the PECARN criteria by Kuppermann et al. (2009)) [3]. Furthermore, while neurological assessments provide an indication on the severity of the head trauma in older children and adults, they are unreliable in infants [5–7]. Thus, it is very important to have specific tools for ciTBI detection in infants.

The gold standard imaging modality to diagnose TBI is head computer tomography (CT) [7]. However, CT exposes patients to ionizing radiation, to which the developing brain of children is particularly vulnerable [8–12]. To reduce the number of CTs, the Pediatric Emergency Care Applied Research Network (PECARN) introduced head injury prediction rules in 2009 [3]. These guidelines recommend close observation instead of CT in the absence of certain criteria [3]. However, while PECARN criteria are useful to identify patients not requiring CT, they lack specificity [13]. Therefore, in clinical reality, the decision to order a CT is still based on individual clinical judgment [14].

An alternative imaging modality without radiation is ultrasound. In infants, the open fontanel and the bone structure allow for cranial ultrasound (CUS) to diagnose intracranial hemorrhage [15, 16]. While routinely used in neonatology, international guidelines do not currently recommend CUS for diagnosis of TBI, claiming insufficient sensitivity [17].

CUS is regularly ordered in the Pediatric Emergency Department at Inselspital University Hospital (Bern, Switzerland) after infant head trauma to assess for the presence of SF and ICH, which both correlate with a higher risk for ciTBI [3, 18]. It is assumed that findings related to ciTBI would show in CUS when performed by a trained radiologist. Yet, this has not been assessed previously. Furthermore, to date, the management of infants with normal CUS is not standardized. Most infants with a normal CUS are observed for up to 24 h as per local guidelines. This allows for a direct comparison of CUS-based diagnoses with the clinical course, as well as additional neuroimaging in certain cases.

The aim of the presented study is to determine the diagnostic accuracy of CUS in detecting SF and/or ICH among infants with minor head trauma. With the hypothesized high accuracy, CUS could give reassurance to reduce the time of clinical observation because of the decreased risk of ciTBI.

## Material and methods

### Study design

This study is a monocentric, retrospective, diagnostic accuracy analysis among infants referred to a pediatric radiology department for CUS after head trauma. The study was approved by the Local Ethics Committee of Bern, Switzerland. Consent was obtained from the parents of all participants included in the study according to ethics proposal.

### Participants

Children younger than 12 months of age referred to the Pediatric Radiology Department of the University Children’s Hospital Bern between July 2013 and August 2020 for CUS after head trauma were included. Eligible infants were identified through a search in the radiology information system. Out of all subjects, those with head trauma as indication for the CUS were selected. Head trauma included falls on the head, blows to the head, high velocity trauma, and unclear events possibly involving trauma to the head. Patients receiving CUS for different reasons, such as suspected meningitis, and patients with known neurological diseases, such as hydrocephalus, were excluded.

Demographic and clinical information was collected from the clinical information system, including PECARN criteria, time interval since the accident, neurological exam, duration of hospitalization (surveillance over 6 h was regarded equal to overnight admission [19, 20]), and final discharge diagnosis. The latter was categorized as concussion (if CUS was normal), SF, ICH, SF with ICH, or other diagnosis.

### Index test

All CUS studies were performed or supervised by a board-certified pediatric radiologist. Siemens Acuson S3000 with a 10-MHz sector transducer and a 9–18-MHz linear transducer was used. The examination included thorough scans in coronal planes starting in the frontal lobes progressing in posterior direction, followed by sagittal and parasagittal planes. In multiple cases, further images were added through axial, transtemporal planes and coronal planes with a linear transducer.

### Reference test

The clinical course during in-hospital surveillance was regarded as reference and considered conspicuous in case of vital sign instability or abnormalities in neurological examination. The rationale is that a ciTBI would show a conspicuous clinical course, respectively ciTBI could be ruled out when an inconspicuous CUS was followed by an uneventful clinical surveillance [3]. Furthermore, where available, CUS findings were compared to CT, MRI, skull radiographs, or follow-up CUS and evaluated for additional information.

### Analysis

The primary outcome was the sensitivity and specificity of CUS as a method to detect abnormalities, which can lead to ciTBI. These included SF and ICH as well as unclear findings with recommendation for further investigation.

Data were collected in REDcap (Nashville, TN, USA). After anonymization, statistical analyses were conducted with Microsoft Excel (Redmond, WA, USA) and R 4.0.2 (Indianapolis, IN, USA). The data does not follow a normal distribution according to the Shapiro test for normality. Therefore, the median values with 25th and 75th quantiles are reported. The Kolmogorov–Smirnov test was used for statistical comparison between different diagnoses, and corresponding *p*-values are indicated. Categorical variables are presented in frequencies and percentages.

For primary outcomes, sensitivity and specificity with confidence interval (CI) [21] and positive and negative predictive values as well as positive and negative likelihood ratios were calculated, respectively.

## Results

**Fig. 1:**
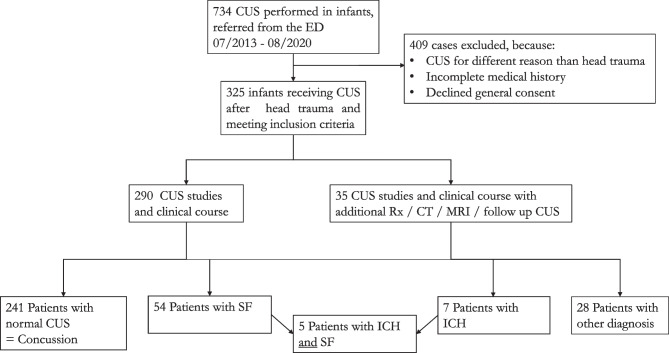
Flow chart, development from the first search to final diagnosis. All of the 325 infants included in the study were observed for several hours, most of them (*n* = 264) for over 6 h up to several days. *CT* computer tomography, *CUS* cranial ultrasound, *ED* emergency department, *ICH* intracranial hemorrhage, *MRI* magnetic resonance imaging, *Rx* skull radiograph, *SF* skull fracture

Between July 2013 and August 2020, a total of 734 CUS in infants were accounted for, whereof 325 patients met the inclusion criteria (Fig. 1).

### Patient characteristics

The characteristics of the study population are listed in Table 1. Additional characteristics can be found in supplementary table 3. To facilitate a comparison between different final diagnoses, columns with the corresponding subdivision were added.

**Table 1:** Characteristics of the study sample. Numbers refer to total numbers of patients, if not declared otherwise. See supplementary table 3 for a more detailed table

		**All patients (*n* = 325) including other final diagnosis (*n* = 28)**	**Patients with normal CUS = concussion (*n* = 241)**	**Patients with SF (*n* = 49)**	**Patients with ICH (*n* = 2)**	**Patients with ICH and SF (*n* = 5)**
History and clinical findings	Median of age (quartile)	**3 months (1–5)**	2 months (1–5)	3 months (2–8)	4 months (2.5–5.5)	3 months (0–4)
	Fall from height (*n*) with median height (quartile)	**248, 70 cm (49–100)**	196, 70 cm (40–100)	41, 80 cm (50–100)	0	5, 120 cm (120–130)
	Other mechanisms of injury*	**77**	45	8	2	0
	Post-traumatic symptoms**	**70**	53	6	1	1
	Skull hematoma occipital, parietal or temporal	**136**	74	48	1	5
	Skull hematoma frontal	**70**	65	1	1	0
Additional imaging modalities	MRI/CT/Rx/follow-up CUS	**35**	7	16	2	5
Clinical course	Neurosurgical intervention	**2**	0	2	0	0
	Surveillance conspicuous	**3**	0	0	1	0
	Duration of hospitalization	**1.4 nights**	0.9 nights	3.2 nights	5 nights	4 nights

The total of patients includes patients with normal CUS, with SF, with ICH, and with SF and ICH, as well as patients with other final diagnosis

*cm* centimeter, *CT* computer tomography, *CUS* cranial ultrasound, *GCS* Glasgow Coma Scale, *ICH* intracranial hemorrhage, *MRI* magnetic resonance imaging, *Rx* skull radiograph, *SF* skull fracture

^*^Blow against head, falling down stairs, car accident, falling out of rolling stroller, shaking trauma, no trauma remembered

**Vomiting, loss of consciousness, altered mental status

Out of a total of 325 patients, 73% (*n* = 241) had a normal CUS and were diagnosed with concussion. SF was found in 17% (*n* = 54), and ICH was diagnosed in seven patients, five of whom showed both ICH and SF. SF was most often parietal (*n* = 25), followed by (parieto-)occipital (*n* = 21), (parieto-)temporal (*n* = 6), and (parieto-)frontal (*n* = 2). ICH were classified as epidural (*n* = 4), subdural (*n* = 2), and intracerebral (*n* = 1). Accordingly, the majority (60%, *n* = 3) of the combined injuries had a parietal fracture with epidural hemorrhage. In 28 cases, a different diagnosis was found. These included cases of suspected non-accidental injury (*n* = 3), as they required special management and neuroimaging (see supplements for complete list of different diagnoses).

The median age of all patients was 3 months. No significant difference in patient age was found for patients with a normal CUS compared to patients with SF and/or ICH (Kolmogorov–Smirnov test, *p* = 0.23). The mechanism of injury was most often a fall from a median height of 70 cm (IQR 49–100 cm). The height of fall in patients with SF and/or ICH was significantly higher (*p* = 0.041). However, the range was remarkable: five SFs were caused by falls from less than 50 cm, and 59 falls from heights over 100 cm caused no SF or ICH. In falls from height, there was a 19% probability (45/248) for SF and/or ICH. This was lower for other mechanisms of injury (10/77 = 13%). Symptoms were rarely reported (22%, 70/325), independent of the diagnosis. On clinical examination, all patients were found to have a GCS score of 14 to 15, and two-thirds of the total sample showed skull hematoma. In cases with SF and/or ICH, skull hematoma was always present, mostly non-frontal.

Additional imaging modalities were rarely ordered: four CT and six MRI examinations were performed. The most often used modality was skull radiography (*n* = 22). Follow-up CUS was performed in 12 cases. Additional neuroimaging was more often performed in patients with SF and/or ICH (23/56 vs. 7/241 in patients with normal CUS).

Neurosurgical intervention was necessary in only two cases, both including impression SF without ICH. The need for intervention was in both cases decided at first evaluation and not due to deterioration during surveillance. The clinical course on the other hand was conspicuous in three other cases, two of which resulted in a different diagnosis (meningitis and convulsion). The third case involved ICH, correctly having been diagnosed in the first CUS. The average length of hospitalization was a median of 1.4 nights for all patients. The latter varied significantly among different diagnoses: while patients with a normal CUS stayed 0.9 nights on average, for SF, the average hospitalization amounted to 3.2 nights, and with an (additional) ICH, the stay extended to 3–7 nights.

### Main outcome

Most of the diagnoses mentioned above were obtained through the first ultrasound and were confirmed with a consistent clinical outcome. The findings on CUS were incorrect in nine cases. Table 2 shows the proportion of false-positive and false-negative results. This corresponds to a sensitivity of 93% ([0.83, 0.97] 95% CI) and a specificity of 98% ([0.95, 0.99] 95% CI). The positive predictive value was 0.91, the negative — 0.99. The calculated diagnostic accuracy was 0.97.

**Table 2:** Proportion of correct and false diagnosis in the CUS. See supplementary table 4 and 5 for further details

**Outcome (by clinical course and/or additional imaging**	**CUS**		
	ICH and/or SF or abnormal finding	Inconspicuous	
ICH and/or SF or abnormal finding	53*	**4**	**Sensitivity = 0.93**
Inconspicuous	**5**	**263**	**Specificity = 0.98**

Positive predictive value = 0.91, negative predictive value = 0.99, positive likelihood ratio = 50, negative likelihood ratio = 0.07, accuracy = 0.97, diagnostic odds ratio = 697. *CUS* cranial ultrasound

*Including one case where CUS showed abnormal findings and not ICH and/or SF: findings suggestive for meningitis, which were then confirmed by lumbar puncture

The four false-negative cases originated from missed SF. No ICH were missed (supplementary table 4). In all five false-positive cases, SF were falsely diagnosed on CUS and could not be confirmed in radiographs or after review of the CUS. Two of these cases were found to have a different diagnosis. A list of all false-positive and false-negative cases can be found in supplementary table 5.

## Discussion

The starting point for this study was the fact that due to the underexplored reliability of CUS, infants after minor head trauma are additionally surveyed over several hours and sometimes undergo additional neuroimaging. The here presented study found a high accuracy of CUS, with a sensitivity of 93% and a specificity of 98% to detect SF and/or ICH. ciTBI is a complication of SF and ICH which need specialist intervention by intensive care or neurosurgical intervention and can therefore be excluded with a high accuracy.

International guidelines on management of head trauma in infants do not recommend CUS, proclaiming a low sensitivity in detecting small, peripheral ICH [17]. Nevertheless, the here presented study finds a high accuracy. Notably, false-positive and false-negative cases did not include any missed ICH but only falsely suspected or missed SFs. This result is in accordance with a study by Masaeli and colleagues [16]. They compared the findings of CUS to head CT and found a sensitivity and specificity of 86% and 98% in detecting ICH in children under 2 years of age. In another study, McCormick et al. tested the accuracy of CUS by letting two radiologists look at CUS images of patients with ICH as well as matched controls without ICH [22]. Herein, the sensitivity varied greatly between the two radiologists, being 50% and 100%, respectively. While the low number of analyzed cases (*n* = 12) limits the reliability of that study, their finding stresses the importance of well-trained radiologists for interpreting CUS, as was the case in the here presented study. Despite trained radiologists and a similar approach to the results presented here, a recent study by Elkhunovich et al. found only moderate sensitivity of 67% for the detection of ICH through CUS [15]. The reasons for the low sensitivity are not clear. Elkhunovich et al. had CT or MRI as a comparison in half of the cases, whereas in the here presented study, additional imaging was available in only 11% of cases. Therefore, one may argue that small ICH, which did not show clinical relevance during observation periods, could have been under-diagnosed on CUS in the presented study. Overall, the here presented results show that CUS is falsely underrated for the diagnostics of ICH in infants. Performed by well-trained radiologists, CUS is therefore suitable and sufficient to rule out clinically relevant ICH.

SF occurred in 54 of 325 cases. There were four cases of missed SFs and five cases of falsely suspected SF, resulting in a sensitivity of 93% and a specificity of 98% for detection of SF through CUS. Several previous studies focused on the diagnostic of skull fracture through point-of-care ultrasound. A recent meta-analysis by Alexandridis showed a sensitivity and specificity of 91% and 96%, respectively, even when performed by emergency residents with only little ultrasound training [18]. While high accuracy in detecting skull fractures could be demonstrated in the study at hand, even more important is the fact that all four missed SF did not correlate with ICH.

The correlation between SF and ICH has been widely discussed. While several studies suggest that SF quadruples the risk of ICH [3, 4, 6, 18], others show that in up to 50% of ICH, no SF can be found [7, 23]. The here presented findings suggest a rather strong correlation: in 71% (5/7) of ICH cases, additional SF was diagnosed. Reciprocally, the risk of ICH in combination with SF was 9% (5/54), whereas general risk of ICH across the study was 2.2% (7/325), corresponding to a fourfold increase in the risk of ICH in the presence of skull fracture. A strong correlation between SF and ICH supports the reliability of an inconspicuous CUS.

Another risk factor for SF and ICH is trauma location. Scalp hematoma on the frontal lobe was associated with SF and/or ICH in only 3% (2/70), while parietal, occipital, or temporal locations increased the risk up to 40% (54/136). These findings are consistent with previous studies, which found 5.6–6 times greater odds of ICH for temporal, parietal, or occipital skull hematoma [23, 24].

Compared to international guidelines, the hospital of focus in this study has a low threshold for hospitalization after head trauma even after unremarkable CT or MRI and a normal neurological examination. Only 19% (61/325) of the patients were discharged within less than 6 h. Average surveillance time in patients with normal CUS was 0.9 nights, for SF 3.2 nights, and for ICH 4.3 nights, even without neurological symptoms. The three cases of deterioration during surveillance included two cases with different diagnoses, unrelated to head trauma, and one case of ICH needing close observation but no neurosurgical intervention. This low number of deterioration is consistent with previous studies [25]. In a systematic review, Donaldson and colleagues suggest that after an isolated linear skull fracture no admission is needed [26]. Admission is associated not only with inconveniences for patients and their parents but also with high healthcare costs.

### Suggested role of CUS and clinical significance

Based on our findings, we propose the use of CUS performed by trained radiologists in inconclusive cases with a moderate risk for SF and/or ICH, e.g., in cases where PECARN criteria recommend a CT, but clinical judgment questions the need for it. Meanwhile, when no CT is recommended according to PECARN criteria, there is neither indication for CUS. Therefore, we suggest that a normal clinical examination and an inconspicuous CUS together with a good instruction for the parents could offer reassurance to reduce the in-hospital surveillance of infants who present to the emergency department after minor head trauma.

### Limitations

In this study, only patients receiving a CUS were included. As it was impossible to determine the number of infants not receiving any imaging study or undergoing directly a CT or MRI, there might be a selection bias. This should be tested in a prospective study. Furthermore, CUS were mostly compared to the clinical outcome. The absence of a second imaging modality in the majority of cases may, thus, lead to overestimating the sensitivity of CUS to detect all SF and ICH. However, ciTBI would have been evident with deterioration during surveillance. Therefore, reported sensitivity includes all clinically relevant SF and ICH, while possibly leaving small SF and fine ICH undetected. Another limitation — not confined to the study — might be the smaller size of the frontal fontanelle in older infants, which would decrease the sensitivity of CUS, especially to detect small peripheral ICH.

During this retrospective analysis, the level of available detail in medical records varied, rendering consistent classification challenging. Late-onset deterioration was assessed by reviewing records from follow-up visits. Parents were instructed to present to the same hospital, in case of deterioration, but presentation elsewhere cannot be excluded.

## Conclusion

This study aimed to determine the diagnostic accuracy of CUS in the detection of SF and/or ICH after minor head trauma in infants, which correlate with ciTBI. The results show a sensitivity of 93% and a specificity of 98%. Furthermore, false-negative cases only involved non-complicated fractures, and no ICH was missed. Therefore, CUS offers a valid option for neuroimaging in cases of moderate risk and gives reassurance to reduce duration of in-hospital surveillance.

## Supplementary Information

Below is the link to the electronic supplementary material.

Supplementary file1 (DOCX 21 KB)

## Data Availability

Original data available from the authors upon request.

## Abbreviations

ciTBI: Clinically important traumatic brain injury
CT: Computer tomography
CUS: Cranial ultrasound
ED: Emergency department
ICH: Intracranial hemorrhage
MRI: Magnetic resonance imaging
PECARN: Pediatric Emergency Care Applied Research Network
SF: Skull fracture
TBI: Traumatic brain injury

## References

[1] Nguyen R, Fiest KM, McChesney J (2016). The international incidence of traumatic brain injury: a systematic review and meta-analysis. Can J Neurol Sci.

[2] Peterson AB, Xu L, Daugherty J, Breiding M (2019) Surveillance report of traumatic brain injury-related emergency department visits, hospitalizations, and deaths. Centers for Disease Control and Prevention, US Department of Health and Human Services. Published online 24

[3] Kuppermann N, Holmes JF, Dayan PS (2009). Identification of children at very low risk of clinically-important brain injuries after head trauma: a prospective cohort study. The Lancet.

[4] Quayle KS, Jaffe DM, Kuppermann N (1997). Diagnostic testing for acute head injury in children: when are head computed tomography and skull radiographs indicated?. Pediatrics.

[5] Greenes DS, Schutzman S (1997). Infants with isolated skull fracture: what are their clinical characteristics, and do they require hospitalization?. Ann Emerg Med.

[6] Schutzman SA, Barnes P, Duhaime AC (2001). Evaluation and management of children younger than two years old with apparently minor head trauma: proposed guidelines. Pediatrics.

[7] Ryan ME, Palasis S, Saigal G (2014). ACR appropriateness criteria head trauma—child. J Am Coll Radiol.

[8] Pearce MS, Salotti JA, Little MP (2012). Radiation exposure from CT scans in childhood and subsequent risk of leukaemia and brain tumours: a retrospective cohort study. The Lancet.

[9] Hall P (2004). Effect of low doses of ionising radiation in infancy on cognitive function in adulthood: Swedish population based cohort study. BMJ.

[10] Brenner DJ, Elliston CD, Hall EJ, Berdon WE (2001). Estimated risks of radiation-induced fatal cancer from pediatric CT. Am J Roentgenol.

[11] Costello JE, Cecava ND, Tucker JE, Bau JL (2013). CT radiation dose: current controversies and dose reduction strategies. Am J Roentgenol.

[12] Kim PK, Zhu X, Houseknecht E, Nickolaus D, Mahboubi S, Nance ML (2005). Effective radiation dose from radiologic studies in pediatric trauma patients. World J Surg.

[13] Lorton F, Poullaouec C, Legallais E (2016). Validation of the PECARN clinical decision rule for children with minor head trauma: a French multicenter prospective study. Scand J Trauma Resusc Emerg Med.

[14] Gerber N, Sookraj K, Munnangi S (2019). Impact of the Pediatric Emergency Care Applied Research Network (PECARN) guidelines on emergency department use of head computed tomography at a level I safety-net trauma center. Emerg Radiol.

[15] Elkhunovich M, Sirody J, McCormick T, Goodarzian F, Claudius I (2018). The utility of cranial ultrasound for detection of intracranial hemorrhage in infants: Pediatric Emergency Care.

[16] Masaeli M, Chahardoli M, Azizi S, Shekarchi B, Sabzghabaei F, Shekar Riz Fomani N, Azarmnia M, Abedi M (2019) Point of Care Ultrasound in Detection of Brain Hemorrhage and Skull Fracture Following Pediatric Head Trauma; a Diagnostic Accuracy Study. Arch Acad Emerg Med 7(1):e53 PMC6905422 31875207

[17] Ryan ME, Pruthi S, Desai NK (2020). ACR Appropriateness Criteria® Head Trauma-Child. J Am Coll Radiol.

[18] Alexandridis G, Verschuuren EW, Rosendaal AV, Kanhai DA (2020) Evidence base for point-of-care ultrasound (POCUS) for diagnosis of skull fractures in children: a systematic review and meta-analysis. Emerg Med J Published online December 3, 2020:emermed-2020–209887. 10.1136/emermed-2020-209887 10.1136/emermed-2020-209887 PMC8717482 33273039

[19] Hamilton M, Mrazik M, Johnson DW (2010). Incidence of delayed intracranial hemorrhage in children after uncomplicated minor head injuries. Pediatrics.

[20] Nigrovic LE, Kuppermann N (2019) Children with minor blunt head trauma presenting to the emergency department. Pediatrics 144(6):e20191495. 10.1542/peds.2019-1495 10.1542/peds.2019-1495 31771961

[21] Mangold S (2013) Evidenzbasiertes Arbeiten in der Physio- und Ergotherapie, Springer-Verlag Berlin Heidelberg. 10.1007/978-3-642-40636-2

[22] McCormick T, Chilstrom M, Childs J (2017). Point-of-care ultrasound for the detection of traumatic intracranial hemorrhage in infants: a pilot study. Pediatr Emerg Care.

[23] Burns ECM, Grool AM, Klassen TP et al (2016) Scalp hematoma characteristics associated with intracranial injury in pediatric minor head injury. Macy ML, ed. Acad Emerg Med 23(5):576–583. 10.1111/acem.12957 10.1111/acem.12957 26947778

[24] Hajiaghamemar M, Lan IS, Christian CW, Coats B, Margulies SS (2019). Infant skull fracture risk for low height falls. Int J Legal Med.

[25] Holmes JF, Borgialli DA, Nadel FM (2011). Do Children with blunt head trauma and normal cranial computed tomography scan results require hospitalization for neurologic observation?. Ann Emerg Med.

[26] Donaldson K, Li X, Sartorelli KH, Weimersheimer P, Durham SR (2019). Management of isolated skull fractures in pediatric patients: a systematic review. Pediatr Emerg Care.

