# Supplementary material for: Minor head trauma in infants — how accurate is cranial ultrasound performed by trained radiologists?
Source: Eur J Pediatr. 2023 Apr 24;182(7):3113–20. doi: 10.1007/s00431-023-04939-9 (PMC10354175; doi:10.1007/s00431-023-04939-9)
Supplement: Supplementary file 1 — Supplementary file1 (DOCX 21 KB) [file 431_2023_4939_MOESM1_ESM.docx]

## Supplements:

**Supplementary Table 3. Characteristics of the study sample**

|  |  |  | **All patients**  **(n = 325) including other final diagnosis (n = 28)** | **Patients with normal CUS (n = 241)** | **Patients with SF (n = 49)** | **Patients with ICH (n = 2)** | **Patients with SF and ICH (n=5)** |
| --- | --- | --- | --- | --- | --- | --- | --- |
| History | Median of Age  (quartile) | | **3 months**  **(1-5)** | 2 months  (1-5) | 3 months  (2-8) | 4 months (2.5-5.5) | 4 months  (0-4) |
|  | Mechanism of injury | Fall from height no  Median height (quartile) | **248**  **70 cm (49-100)** | 196  70 cm (40-100) | 41  80 cm (50-100) | 0  (-) | 5  120 cm (120-130) |
|  |  | Falling down stairs | **10** | 9 | 0 | 1 | 0 |
|  |  | Blow against head | **36** | 30 | 3 | 1 | 0 |
|  |  | (possible) Shaking trauma | **5** | 0 | 0 | 0 | 0 |
|  |  | Higher velocity trauma* | **11** | 5 | 0 | 0 | 0 |
|  |  | No trauma remembered | **15** | 1 | 5 | 0 | 0 |
|  | Interval since trauma | Same day | **288** |  |  |  |  |
|  |  | The day before | **17** |  |  |  |  |
|  |  | earlier | **20** |  |  |  |  |
|  | Symptoms | Vomiting, LOC, AMS | **70** | 53 | 6 | 1 | 1 |
| Clinical findings | GCS | 15 | **317** |  |  |  |  |
|  |  | 14 | **8** |  |  |  |  |
|  | Skull Haematoma | none | **119** | 102 | 0 | 0 | 0 |
|  |  | Occipital, parietal, temporal | **136** | 74 | 48 | 1 | 5 |
|  |  | frontal | **70** | 65 | 1 | 1 | 0 |
| Imaging modalities | CUS (additional) | | **12** | 1 | 4 | 1 | 5 |
|  | Rx | | **22** | 6 | 13 | 0 | 1 |
|  | CT | | **4** | 0 | 2 | 1 | 0 |
|  | MRI | | **6** | 0 | 2 | 2 | 0 |
| Clinical course | Neurosurgical intervention | | **2** | 0 | 2 | 0 | 0 |
|  | Surveillance conspicuous | | **3** | 0 | 0 | 1 | 0 |
|  | Duration of hospitalization | | **1.4 nights** | 0.9 nights | 3.2 nights | 5 nights | 4 nights |
| CUS sufficiency | PECARN recommends CT | | **63** |  |  |  |  |
|  | CUS sufficient | | **296** |  |  |  |  |
|  | Additional imaging without new findings | | **20** |  |  |  |  |
|  | Additional imaging with new findings | | **9** |  |  |  |  |
| *car accident, falling out of rolling stroller  AMS = altered mental status, CT = computer tomography, CUS = cranial ultrasound, GCS = Glasgow Coma Scale, LOC = loss of consciousness, MRI = magnetic resonance imaging, Rx = skull radiograph | | | | | | | |

**Estimated heights**

Wherever height of fall was not documented in centimeter, it was estimated according to average values:

- parent’s arm: 120 cm
- changing table: 90 cm
- troller: 70 cm
- Tripptrapp (high chair for children): 70 cm
- child’s height: 50 cm

**Other diagnosis (n=28):**

- 9 Car accident, fall out of house or other fall without head involvement
- 4 unclear haematoma or petechiaeon head
- 4 epilepsy or convulsion
- 4 unclear event or unclear vigilance disturbance
- 3 Birth trauma
- 3 (suspicion of) battered child
- 1 meningitis
- 1 gastroenteritis

### Statistics

Age: no normal distribution, therefore use of Kolmogorov-Smirnov Test.

concussion vs SF and/or ICH p=0.23 -> No significant age difference

Height of fall: no normal distribution, therefore use of Kolmogorov-Smirnov Test.

- concussion vs SF+/-ICH, p= 0.053 -> not significant difference
  - Median (Quartile) of SF +/-ICH: 100 cm (50-120)
- concussion vs SF, p=0.33 -> no significant difference.
- concussion vs ICH(+SF) p= 0.0041 -> significant

**Supplementary Table 4. CUS accuracy subdivided according to diagnosis**

|  | CUS conspicuous | | CUS inconspicuous | |
| --- | --- | --- | --- | --- |
|  | **correct** | **incorrect** | **correct** | **incorrect** |
| Normal CUS = concussion | **0** | **3** | **238** | **0** |
| SF | **45** | **0** | **0** | **4** |
| ICH and SF | **5** | **0** | **0** | **0** |
| ICH | **2** | **0** | **0** | **0** |
| Other diagnosis | **1** | **2** | **25** | **0** |
| **Sensitivity** for fracture diagnosis: 50/54* = 93%.  **Sensitivity** for detection of intracranial haemorrhage:  7/7* = 100%  * 3 cases with SF known beforehand and 1 case of ICH known beforehand are excluded in calculation  CUS = cranial ultrasound, ICH = intracranial haemorrhage, SF = skull fracture | | | | |

**Supplementary Table 5. False negative and false positive ultrasound findings**

| **Case no** | **Age (month)** | **US finding** | **CT/MRI/skull radiograph finding** |
| --- | --- | --- | --- |
| No 40 | 3 | False negative | Rx radiograph showed parieto-temporal fracture |
| No 116 | 0 | False negative | Radiograph showed parieto-occipital fracture |
| No 323 | 2 | False negative | Follow up ultrasound showed parietal fracture |
| No 401 | 8 | False negative | Radiograph showed a parietal fracture |
| No 146 | 3 | False positive | Follow up ultrasound could not confirm the suspected fracture |
| No 306 | 10 | False positive | CUS found a fracture haematoma, the fracture could not be confirmed in radiograp. Due to unclear history of accident and clinic, final diagnosis was no TBI. |
| No 333 | 0 | False positive | Radiograph did not confirm suspected fracture |
| No 376 | 2 | False positive | Review of the CUS during hospitalization found initial susptected signs of fracture as wrong. |
| No 394 | 0 | False positive | Radiograph could not confirm fracture. Final diagnosis was no TBI but birth trauma. |
